# Speculations on the evolution of humoral adaptive immunity

**DOI:** 10.1111/imcb.12323

**Published:** 2020-03-25

**Authors:** Miles B Horton, Edwin D Hawkins, Susanne Heinzel, Philip D Hodgkin

**Affiliations:** ^1^ Division of Immunology The Walter and Eliza Hall Institute of Medical Research Parkville VIC 3052 Australia; ^2^ Department of Medical Biology The University of Melbourne Parkville VIC 3010 Australia

**Keywords:** B cells, cell fate, cell signaling, comparative immunology, evolutionary immunology, humoral immunity, proliferation

## Abstract

The protection of a multicellular organism from infection, at both cell and humoral levels, has been a tremendous driver of gene selection and cellular response strategies. Here we focus on a critical event in the development of humoral immunity: The transition from principally innate responses to a system of adaptive cell selection, with all the attendant mechanical problems that must be solved in order for it to work effectively. Here we review recent advances, but our major goal is to highlight that the development of adaptive immunity resulted from the adoption, reuse and repurposing of an ancient, autonomous cellular program that combines and exploits three titratable cellular fate timers. We illustrate how this common cell machinery recurs and appears throughout biology, and has been essential for the evolution of complex organisms, at many levels of scale.

## Graphical abstract

We argue that the development of adaptive immunity resulted from the adoption, reuse and repurposing of an ancient, autonomous cellular program. Signature features of this ancient program can be seen exploited in many arms of the immune response.

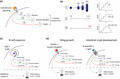

## Looking Back in Time

There are signatures and ghostly remnants of events long past carried in the genes and cellular processes that fuel the contemporary biosphere. Some events, such as the cellular adoption of mitochondria, are permanent foundations, and reveal to us the striking consequence of a distant fortuitous symbiosis.^1^ We are now used to finding other examples of flexible, adaptable biological processes that are tweaked and tuned and reused in a myriad of ways in different species. With care, these origins and paths can be identified with the many modern tools available. What we find now is helping to expose this connected history and to complete the detailed story of evolution. Of course, this works in reverse and evolution can serve as a powerful organizing principle to try to understand the current complex state and how it came to be.^2^

The development of immunity is a potent force for gene selection.^3^, ^4^, ^5^ In most species, immune genes are some of the most active and pliable. Even closely related species can show significant divergence in immune genes, presumably as a result of chance decisions and local, or isolated, selection forces.^6^, ^7^, ^8^ Despite the complexity, shadowy evolutionary signatures and clues to the past are emerging rapidly and offer major new insights into the cellular machinery underlying immunity.

## A Brief Overview of the Innate to Adaptive Junction

The field of comparative adaptive immunology has been recently galvanized by the discovery of two distinct somatic diversification mechanisms for the generation of antigen receptors. The two mechanisms are found at the junction between jawless vertebrates, the lamprey and hagfish, and jawed vertebrates. Until recently, the more primitive jawless vertebrates were thought to be devoid of an adaptive system, whereas the jawed vertebrates deliver almost fully formed, familiar T‐ and B‐cell systems.^9^, ^10^, ^11^ Adaptive systems in jawed vertebrates utilize randomization of immunoglobulin (Ig) superfamily genes and the selection of multiple cell types during active immune responses. Thus, it appeared that chance Ig gene family–dependent events in the vertebrate lineage uniquely enabled the cell response patterning necessary for a complete adaptive immune outcome. However, this dogma has now been rewritten.^10^, ^12^, ^13^, ^14^, ^15^, ^16^ We now know that the agnathan lampreys developed a parallel adaptive system, with equivalent cellular selection features but altered receptors based on Toll‐like receptor (TLR) genes. This discovery indicates that the cellular principles were in place for adaptation in lamprey jawless vertebrates, and the actual receptors and substrates for solving the binding and combinatorial problems could have multiple answers. It is now clear that the evolving systems found the capacity for programming cell autonomous selection and expansion, and likely cooperation. Where did it find this program? It seems unlikely to have been invented twice *de novo*. Rather, we argue, it was there all the time, within the multicellular genetic coding, fully adaptable and pliable for this new purpose. We review briefly the cellular machinery for cell development, programming and patterning, and then speculate on the key steps in the innate to adaptive transition that corralled this machinery for an entirely new purpose.

## The Autonomous B‐Cell Program

When studying vertebrate immunity, it is hard to avoid being overwhelmed by its apparent complexity. Lymphocytes are surrounded by finely detailed microenvironments and express a large number of receptors and continually process signals that influence their viability, fate and position. Thus, cells of adaptive responses are highly mutable and continually regulated throughout their lifetimes. The B cell undergoes numerous such events before making antibody of different types. As circulating mature follicular cells, they do not secrete antibody but must be activated to undergo extensive proliferation, to change antibody type and differentiate into short‐ and long‐lived antibody‐secreting (plasma) cells.^17^ However, when studied in isolation, when complex signals are stripped away, we see evidence for simple behaviors that have all the hallmarks of primitive autonomous programs that have recurred in biology and are found repeatedly in different forms. These features appear in many versions of adaptive responses, and we believe these provide an echo of their evolutionary history.

**Figure 1:**
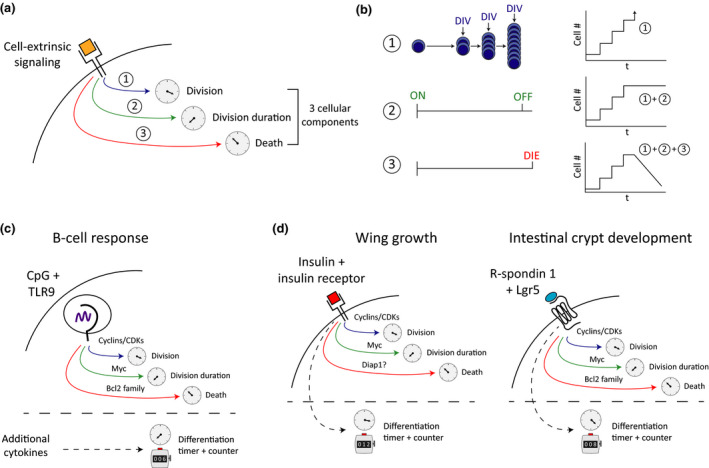
Three ancient cell fate timers combine for ubiquitous tissue patterning outcomes. **(a)** The basic program of the 3‐TM. Engaging the receptor (or receptors) motivates separate modular cellular changes that initiate (1) division; (2) the duration of division and (3) the time to die. Note that the times for 2 and 3 can be inherited and passed to progeny. **(b)** The net effect of each timer applied in sequence, leading to the automatic increase and timed loss of the stimulated cell and subsequent clonal family over time. **(c, d)** These diagrams show how similar autonomic mechanical features underlie the B‐cell response, insect wing development and the growth of the intestinal crypt. Additional modular cell fate programs that interleave with 3‐TM are represented below the dashed line. These may include the integration of further signals as in **c**, or may function as additional independent modules that regulate differentiation fates. These fates may function as timers, as with the components of the 3‐TM, or could use cell division as a “counting” mechanism, with a threshold number of divisions triggering a cell fate decision. 3‐TM, three‐timer model; CDK, cyclin‐dependent kinase; cell #, cell number; DIV, division; t, time.

A clear example of such a program comes from the follicular B cell’s autonomous proliferative response to the TLR9 ligand, CpG unmethylated DNA.^18^ This response has been extensively filmed and analyzed to reveal its remarkable features. It is mediated by nuclear factor kappa‐light‐chain‐enhancer of activated B cells (NF‐κB) signaling that results in stimulated cells that divide three or four times, before they return to a small, nondividing state and then die. The cells do not differentiate into antibody‐secreting cells unless other signals are provided.^18^, ^19^, ^20^ Thus, lymphocytes are capable of implementing a program of activation, asynchronous proliferation, cessation and death in response to the presentation of a single stimulus. Quantitative studies of the CpG response program indicate that the net outcome is the result of three timed fates initiated by activation, subsequently referred to as the three‐timer model (3‐TM).^19^, ^20^, ^21^ The first timer controls time between divisions while the second timer dictates the time allowed for the series of cell divisions before returning to quiescence (the cell’s division destiny, heritable from the initial cell). Finally, the third timer regulates the time to die, which, for CpG‐stimulated B cells, is also heritable from the founding cell and is unaffected by cell division.^22^ Molecular regulation of the first timer is attributable to cyclins and cyclin‐dependent kinases.^19^ The second timer is strongly coupled to expression of the transcription factor Myc, protein levels of which accumulate and fall in cells over time and are also unaffected by division.^22^ The timing of cell death (third timer) is controlled by the net effect of multiple proapoptotic and antiapoptotic molecules.^22^, ^23^ The components and mechanism of the 3‐TM are illustrated in Figure 1a, b to highlight how it leads to a canonical immune response pattern. As a result of the absence of the generation of any additional specialized cell types, as well as the ancient origin of TLRs,^24^, ^25^ it appears that the TLR9‐induced B‐cell program provides a window back to simpler organisms that relied on innate immune recognition to initiate protective responses that could include limited cell expansion.

This simple response sheds light on how an ancestral program for limited cell‐intrinsic proliferation, cessation and cell loss was adopted to enable the emergence of adaptive immunity. Evidence for such an evolutionary legacy is provided by the conservation of intracellular events coupled to TLR activation across diverse evolutionary relationships. TLR‐induced activation of the NF‐κB pathway, for instance, is shared between mammals, insects and sea urchins.^24^, ^25^, ^26^ Furthermore, there is evidence to suggest that the molecular control of the 3‐TM outlined previously is evolutionarily ancient. Myc and its homologs have important roles in controlling proliferation in stem cell and gamete populations in the metazoan *Hydra*.^27^, ^28^ The control of programmed cell death by the Bcl‐2 family proteins is likewise significantly evolutionarily conserved, with homologs identified and extensively studied in *Caenorhabditis elegans*.^29^, ^30^

Of course, for most T‐ and B‐cell responses, the cellular behaviors are significantly more complex than that seen for isolated, CpG‐stimulated B cells. This is a consequence of the imposition of three further advanced control features that presumably evolved later, and further interleaved with the basic 3‐TM program, to achieve a greater degree of control. These advanced control features are (1) to modify and diversify the signals that trigger the response program; (2) to incorporate differentiation events, often linked to division progression, that allocate responding cells to a range of effector outcomes and diversify the immune response and (3) to develop three‐dimensional tissue channeling and cell interaction systems to optimize antigen detection, signal integration, cell selection and cooperation. It is the simultaneous actions of these interleaved cell modules that underpin why the vertebrate immune system is extremely complex.^31^, ^32^ Nevertheless, despite the additional layers of regulation, the core adoption of the 3‐TM that utilizes regulable timers based on cyclins, Myc and death molecules or their homologs appears common to all.

## Autonomous Programming in Developmental Biology

We find it striking that features of the molecular and cellular origins of cell fate patterning that drives adaptive immune responses can be found in many systems under study in developmental biology. Many of the desired outcomes are shared across the two disciplines. The generation of cellular diversity is a critical step in a successful immune response. The generation of sufficient cell numbers and the partitioning of functions among the generated cells are complex tasks that must be carried out with great precision. Too many, or too few of a particular cell type could be catastrophic to the chances of an organism’s survival during an infection. This is analogous to the processes of cellular diversification carried out during development, with similar consequences coupled to potential unsuccessful outcomes. Therefore, here we highlight how a common autonomous program underpins the generation of cellular diversity and is shared across multiple biological processes such as embryonic development, tissue homeostasis and adaptive immunity. Following are some notable examples of complex cellular systems that share the common principles of autonomous cellular programming coupled to asynchronous proliferation, differentiation and cell death. These examples demonstrate that carried within cells is the genetic instructions for (1) patterning tissue, (2) growth and limiting the total level of this expansion and (3) clearing cells by apoptosis. These three controls are likely used in different ways, in many examples of tissue formation, in body patterning and in initiating and maintaining cell types. These three controls are therefore ready to be copied and incorporated for immunity, and likely explain why cyclins, Myc and death molecules or their homologs underpin so many cellular systems throughout evolution.

## Making a Wing Has Analogous Features to an Adaptive Immune Response

Organ growth requires precise control of cellular proliferation and patterning, as well as of growth cessation. The imaginal discs of the developing *Drosophila* wing is a well‐studied system for investigating the molecular control and cellular principles involved in the development of a complex tissue.^33^ At the beginning of the transition from the larval to the adult stage, the imaginal disc comprises a small number of precursor cells poised to undergo multiple rounds of cell division.^34^ Rapid cell division is initiated by external signaling through the insulin/insulin‐like growth factor pathway and is further regulated by the disc‐intrinsic production of the Dpp signaling molecule, ultimately resulting in an upward of 1000‐fold expansion in cell number over 4 days of larval development.^33^, ^35^ These signals promote an intracellular signaling response that drives cell growth and cell cycle progression. This program is strongly linked to the proto‐oncogene Myc, which transcriptionally regulates essential components of cell division and growth, including ribosomal activity and metabolic pathways.^35^, ^36^ Individual proliferating cells compete with each other based on their levels of Myc. Myc‐overexpressing cells proliferate more extensively than their wild‐type counterparts, and Myc‐deficient cells are correspondingly outcompeted. After 4 days of growth, the cells of the wing disc will stop proliferating as the wing reaches its final size. The process of growth arrest appears to be intrinsic to the proliferating cells of the wing disc, as they will stop dividing even in the presence of ongoing signaling and an extended period of larval development.^33^ However, unlike the cessation of division in the humoral immune response, this is achieved by the extension of cell cycle length, rather than an exit from division progression.

## Further Analogs: Autonomous Organoid Aggregation

Related and parallel autonomous cell development can be found in other complex organs, previously thought to be dependent on high levels of tissue organization. The mammalian intestinal epithelium requires continuous, rapid self‐renewal to maintain mucosal barrier integrity and function. This tissue is organized into distinct functional units consisting of a luminal villus, predominantly composed of mature enterocytes and Paneth cells, and a crypt, which contains about six Lgr5^+^ stem cells that are continuously giving rise to new cell types. In studies that made a significant contribution to the development of organoids, the Clevers laboratory established that single Lgr5^+^ crypt stem cells could generate structures resembling intestinal tissue in the presence of minimal growth signaling provided by the Wnt pathway ligand, R‐spondin‐1.^37^ The generation of these structures from single cells was highly reproducible, the structures contained all mature cell types of the intestinal epithelium, and they could be maintained in culture long term (at least 8 months). The lumen of these organoid structures contained the postapoptotic remains of enterocytes that had undergone cell death and budded from the tips of villi, reminiscent of the turnover of epithelial cells *in vivo*. Furthermore, only the Lgr5^+^ stem cells displayed evidence of Wnt pathway activity (nuclear β‐catenin), suggesting that the emergence of distinct cell types, their proliferation rates and the timing of apoptosis are established early in the cellular hierarchy and inherited through multiple rounds of cell division.

Additional work has highlighted the role of the Myc pathway in controlling proliferation of intestinal crypt stem cells, acting as an important transcriptional target of Wnt/β‐catenin signaling, and translating the activity of this pathway into the control of cell division.^38^, ^39^, ^40^ This is supported by studies showing that the absence of the cofactors required for Myc expression in crypt stem cells impairs their capacity for regeneration *in vivo*.^38^, ^39^, ^40^ Furthermore, analysis of apoptosis components within intestinal tissues across mouse and human revealed that high expression of antiapoptotic Bcl‐2 is restricted predominantly to the Lgr5^+^ stem cells of the crypt base, and expression falls gradually as cells progress toward the villus tip.^41^ This raises the potential for a timed loss of antiapoptotic molecule expression as cells mature, ultimately resulting in their programmed death after their terminal differentiation into enterocytes at the villus tip.

Together, these data demonstrate a remarkable capacity for mature stem cells to autonomously generate cellular diversity concurrent to rapid cell division without the need for external instructive signaling to control the fate decisions of individual cells. We speculate that these are common properties that are shared in the adaptive immune system, and the capacity for the cell‐intrinsic generation of complex cellular outcomes in response to restricted signaling events closely resembles an adaptive immune response. Thus, we argue that these cellular programs share, build upon and are further examples of a common evolutionary origin. Consequently, we reason that it is highly likely that the behavior of intestinal crypt stem cells could be accurately recapitulated by probabilistic modeling incorporating the timers of division motivation, division rate and the onset of cell death in a manner similar to modeling of T‐ and B‐cell responses. These parallels that might be traced to molecular patterning timers in each of our examples are illustrated in Figure 1. Our figure also illustrates how this core 3‐TM cell machinery controlling cell generation, cessation and loss, can be interleaved with additional modular division‐dependent and timed differentiation programs to diversify and complete the tissue patterning outcomes.

## Building an Immune System from Modular Cell Response Programs

The considerations discussed previously, the parallels in autonomous programming and their evolutionary origins, point to an ancient biological mechanism. This ancient mechanism brings together three controls for fate and tissue patterning (division, division progression and death) that can be placed under the control of different initiating receptors (or sets of receptors) depending on the biological context. This could enable the pattern of net outcome, such as the total cell number generated and their longevity, to be manipulated and optimized.

Given this context, we argue that the solution needed for adaptive immunity, of finding and selecting cell clones, can be viewed as modular and evolvable as it can utilize readily available cell functional units, including each of the 3‐TM cellular programs exemplified by the CpG B‐cell response. This understanding of somatically diversified antigen receptors superimposing over a pre‐existing cellular program fits well with the observation of convergent evolution of adaptive immunity in the jawed and jawless vertebrates. In the lamprey and hagfish, this cellular programming is connected to variable lymphocyte receptor family genes, which are predominantly composed of the leucine‐rich repeat regions that are essential to the function of TLRs. Whereas in the jawed lineage, the equivalent selective pressures lead to connecting the Ig family to a similar autonomous program. Clearly, although the molecular strategies utilized by the two phylogenies are distinct, both are layered on top of a common set of lymphoid cell types and their corresponding response genes. This provides strong evidence that these cells and their programming preceded the emergence of a fully fledged adaptive immune system.^10^, ^11^ As such, we speculate that the responses of VLRA+ and VLRB+ lymphocytes in the jawless vertebrates would share the same cellular principles and molecular control of T and B cells in the jawed vertebrates, including Myc‐dependent proliferation bursts and Bcl‐2 family protein‐dependent, timed apoptosis.

In the following sections, we examine in more detail the possible origins for how the broad recognition of foreign material by innate receptors became coupled to the 3‐TM that modulates the adaptive cellular responses. Not surprisingly, the development of receptors and defense strategies that were drawn into the new adaptive programs were also sourced from available substrates that were already well invested in the immune defenses of more primitive organisms.

## Early Origins of TLR Responses and a Cellular Blueprint in *Drosophila*

A very early evolutionary example of innate receptor development is in the primitive organisms that comprise the invertebrate deuterostome lineage, in which functional TLR genes have been identified. The genome of the sea squirt *Ciona intestinalis* contains two identified TLRs: Ci‐TLR1 and Ci‐TLR2, consisting of intracellular Toll/interleukin‐1 receptor and extracellular leucine‐rich repeat region domains.^24^, ^25^ These receptors are triggered by multiple bacterial components, including cell walls and flagellin, as well as by poly‐I:C. The binding of TLRs to these pathogen‐associated molecular patterns initiates signaling cascades involving molecules conserved in vertebrate immune systems, such as MyD88 and NF‐κB, and triggers the release of effector molecules including tumor necrosis factor‐α and antimicrobial peptides.^24^, ^25^, ^26^

During the course of evolution, the established autonomous cellular programming that was essential to the development of complex multicellular organisms intersected with immune signaling to generate a primitive cellular immune response. Evidence for such a primitive cellular program from which mammalian lymphocyte behavior may have emerged can be found in the responses of the innate immune system of insects, such as *Drosophila melanogaster.* ^42^, ^43^, ^44^Along with other insects, *Drosophila* do not possess an adaptive immune system, and instead rely on an innate immune response carried out by a population of phagocytic cells known as hemocytes. Hemocytes comprise the bulk of the cellular immune system in insects and are categorized into three distinct lineages: plasmatocytes, lamellocytes and crystal cells. These cells develop from a specialized lymphoid organ, known as the lymph gland, and circulate throughout the *Drosophila* hematopoietic system in a quiescent state. Although lacking antigen receptors, hemocytes recognize foreign microbial material via innate pattern recognition receptors and undergo an immune response consisting of proliferation, differentiation and effector functions.^45^, ^46^ Depending on the nature of infection, these cells will either terminally differentiate into phagocytic plasmatocytes to engulf foreign microbes, or they will acquire the phenotype of lamellocytes to encapsulate multicellular parasites.

The activation of hemocytes involves the binding of nonself‐material by surface pattern recognition receptors, most notably the Toll receptor, which triggers an intracellular signaling cascade resulting in cellular proliferation and differentiation. Many of the signaling receptors and pathways involved in hemocyte responses are conserved in mammalian humoral immunity, including in both T‐dependent and T‐independent B‐cell responses, such as TLR, Janus kinase/signal transducers and activators of transcription and mitogen‐activated protein kinase signaling.^44^, ^46^ Furthermore, as in mammalian B cells, disruption of the genes involved in these pathways results in overgrowth of hemocytes and the development of hematological malignancies.^47^, ^48^

## *Drosophila* Dscam—a Primitive Antibody

In addition to their capacity for proliferation and differentiation, hemocytes also possess a restricted repertoire of an adhesion protein, Down syndrome cell adhesion molecule (Dscam). Dscam is a member of the Ig superfamily. Approximately 18 000 distinct isoforms of the cell surface, membrane‐bound Dscam are generated in *Drosophila* by alternative splicing of multiple Ig domains.^49^, ^50^ While bound to the hemocyte cell surface, Dscam molecules recognize and bind foreign material and facilitate the process of phagocytosis. Dscam is also secreted by hemocytes and circulates throughout the *Drosophila* hemolymph, binding to bacterial components and opsonizing them for phagocytosis. Dscam molecules are highly conserved across insects, and a study of *Anopheles gambiae* AgDscam revealed that the isoform repertoire is responsive to distinct infections, demonstrating ongoing feedback driving the selection of isoforms with specific adhesive characteristics.^51^ Furthermore, increased AgDscam‐binding affinity was observed when produced by cells previously exposed to the target pathogen.^51^

Therefore, the invertebrate immune system utilizes a somatically diversified receptor which is coupled to a cellular response and subject to ongoing feedback. Thus, the immune response of invertebrates may represent an evolutionary blueprint for the cellular and molecular program that has been developed in mammalian humoral immunity. Although the cellular immune response in invertebrates has not been as completely described as in mammals, the conservation of basic cellular functions, signaling pathways and effector molecules would suggest that there are many features described in humoral immunity that are identifiable in the invertebrate cellular immune response. We speculate that the cellular principles underpinning the hemocyte response mirror those of follicular B cells, whereby asynchronous activation, proliferation and differentiation of hemocytes leverage the principles of cell autonomy and stochasticity to guarantee successful outcomes to infection.

## The Coexistence and Optimization of Primitive and Advanced Strategies

We now ask what roles CpG/TLR9‐based pathways play when more sophisticated antibody production systems, that include T‐dependent pathways and germinal centers (GCs), that can improve affinity exist in the same host? In answering, we note even more examples of primitive humoral responses in the vertebrate immune system. For instance, B‐1 cells that differentiate and produce a relatively limited repertoire of natural antibody, usually IgM, without priming, are mimicking the action of simple humoral agglutinin production by invertebrates and most multicellular organisms.^52^ This coexistence of more primitive B‐1 cells is likely affected by thermodynamic features governing antibody binding to targets in solution that determines how large a repertoire of receptors is needed for an effective humoral immune response. These constraints dictate that a combination of strategies are needed to minimize the time to protection against antigens comprising epitopes of multiple, distinct valences.^53^ Fitting with this concept, T‐independent B‐cell stimuli are strongly synergistic with cross‐linking B‐cell receptor ligation, promoting development of antibodies associated with bacteria that also have a high epitope valence.^54^ Thus, the more primitive modes of antibody regulation act quickly and typically target multivalent epitopes. These pathways to antibody complement the more sophisticated T‐dependent responses that can achieve high affinity against monovalent antigens but take a longer time to be selected.

## 3‐TM Features Are Seen in the GC

**Figure 2:**
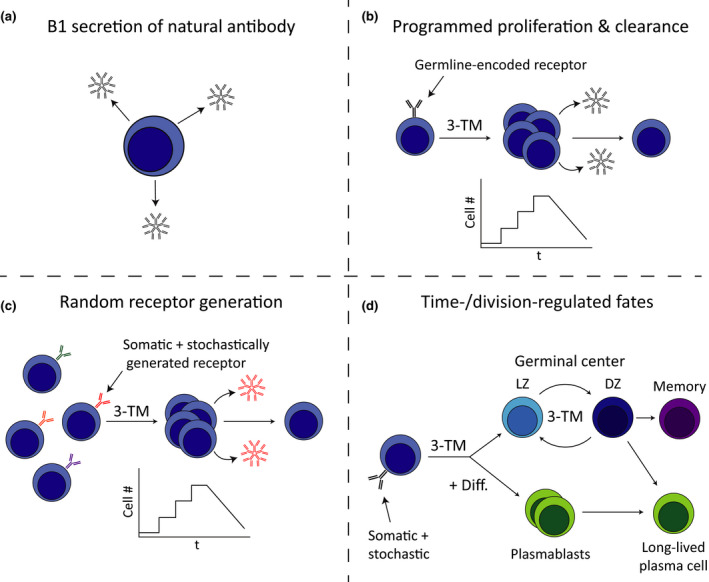
Key steps in humoral immune cell evolution. **(a)** Simple intrinsic secretion of natural antibodies or agglutinins into fluid spaces for B‐1 cells in vertebrates, but also observed for agglutinins, defensins and lectins in other, more primitive species. **(b)** Germline‐coded receptors of varying degrees of diversity trigger a limited expansion and therefore increased production of secreted material but with minimal further diversification of effector activity. This is seen for the CpG response in vertebrates, but also for hemocyte activation and contraction in insects, including stochastic splicing to diversify and select Dscam protein variants by *Drosophila*. **(c)** Diversification of receptor and coupling to the same 3‐TM limited, intrinsic response program. **(d)** Coupling of further effector fate changes to 3‐TM responses by time and/or division to diversify and engage selected specificities with alternative immune strategies. This latter set of programs can be subject to a high degree of feedback and further cell signaling to fine‐tune and optimize the effective outcomes. Some activated cells also enter and seed the germinal center response and undergo repeated cycles analogous to 3‐TM expansion and loss that include timed Myc‐dependent proliferation initiated by B‐cell receptor and T‐cell signals, with cells shuttling between light zone (LZ) and dark zone (DZ) regions and this results in the selection of high‐affinity cells. 3‐TM, three‐timer model; cell #, cell number; Diff, differentiation; t, time.

Recent studies of GC reactions suggest that the components of the 3‐TM play a key role in its regulation. In the GC B cells undergo successive rounds of selection and stimulation, shuttling between light and dark zones as centrocytes and centroblasts and undergoing extensive receptor mutation.^55^, ^56^ This proliferation of GC B cells is tightly controlled by coupling of an Myc‐dependent division timer to B‐cell receptor selection, whereby the highest affinity cells in the GC light zone receive the strongest external signaling, thus providing them with a substantial duration in which to divide within the GC dark zone.^57^, ^58^, ^59^ This extension of proliferation promotes selection of the most effective clones to undergo further somatic hypermutation.^60^ The process is also balanced by a dynamic death timer that functions to prevent continuous accumulation of rapidly dividing GC cells.^59^, ^61^, ^62^ The timed transition of GC cells between centroblasts and centrocytes may also represent an additional component integrated into the GC 3‐TM.^63^ This model of GC behavior thus demonstrates that the basic principles outlined in the 3‐TM can be integrated into complex, ongoing processes and complemented by additional layers of regulation, such as intercellular communication, cell migration and competition. A summary of our projected key steps in the progressive modification and evolution of humoral immune response, highlighting the modular, repeated nature of the recurring cellular programs, is presented in Figure 2.

## Looking Ahead

Our motivation for writing this speculative article was to search for links between an emerging modular view of immune cell control and its evolutionary past. We focused on the tripartite timers revealed by CpG‐activated B cells that motivate a simple program of expansion, cessation and clearance, and found evidence for its ancient origins and adaptability for different purposes. We anticipate that attempting to trace and isolate the steps in cell evolution and how functional modules have been captured and utilized by immune cells can further inform and help guide our interpretation of the operation of the very complex vertebrate immune system. While intellectually appealing for our understanding, we also believe that finding these connections in further detail can instruct and inform the building of hierarchical, multiscale models that can interpret complex signal sensing problems and explain the mysterious logic of the immune response.

## Conflict of Interest

The authors have no financial conflicts of interest.

## References

[1] Sagan L . On the origin of mitosing cells. J Theor Biol 1967; 14: 255–274. 11541392 10.1016/0022-5193(67)90079-3

[2] Behrman EL , Howick VM , Kapun M , *et al* Rapid seasonal evolution in innate immunity of wild *Drosophila melanogaster* . Proc Biol Sci 2018; 285. 10.1098/rspb.2017.2599 PMC5784205 29321302

[3] Fumagalli M , Sironi M , Pozzoli U , Ferrer‐Admetlla A , Pattini L , Nielsen R . Signatures of environmental genetic adaptation pinpoint pathogens as the main selective pressure through human evolution. PLoS Genet 2011; 7: e1002355. 22072984 10.1371/journal.pgen.1002355 PMC3207877

[4] McTaggart SJ , Obbard DJ , Conlon C , Little TJ . Immune genes undergo more adaptive evolution than non‐immune system genes in *Daphnia pulex* . BMC Evol Biol 2012; 12: 63. 22577801 10.1186/1471-2148-12-63 PMC3457901

[5] Waterhouse RM , Kriventseva EV , Meister S , *et al* Evolutionary dynamics of immune‐related genes and pathways in disease‐vector mosquitoes. Science 2007; 316: 1738–1743. 17588928 10.1126/science.1139862 PMC2042107

[6] Crawford JE , Guelbeogo WM , Sanou A , *et al* De novo transcriptome sequencing in *Anopheles* funestus using Illumina RNA‐seq technology. PLoS One 2010; 5: e14202. 21151993 10.1371/journal.pone.0014202 PMC2996306

[7] Erler S , Lhomme P , Rasmont P , Lattorff HM . Rapid evolution of antimicrobial peptide genes in an insect host‐social parasite system. Infect Genet Evol 2014; 23: 129–137. 24530902 10.1016/j.meegid.2014.02.002

[8] Chavez‐Galarza J , Henriques D , Johnston JS , *et al* Signatures of selection in the Iberian honey bee (*Apis mellifera iberiensis*) revealed by a genome scan analysis of single nucleotide polymorphisms. Mol Ecol 2013; 22: 5890–5907. 24118235 10.1111/mec.12537

[9] Flajnik MF , Kasahara M . Origin and evolution of the adaptive immune system: genetic events and selective pressures. Nat Rev Genet 2010; 11: 47–59. 19997068 10.1038/nrg2703 PMC3805090

[10] Boehm T , Hirano M , Holland SJ , Das S , Schorpp M , Cooper MD . Evolution of alternative adaptive immune systems in vertebrates. Annu Rev Immunol 2018; 36: 19–42. 29144837 10.1146/annurev-immunol-042617-053028

[11] Boehm T . Design principles of adaptive immune systems. Nat Rev Immunol 2011; 11: 307–317. 21475308 10.1038/nri2944

[12] Guo P , Hirano M , Herrin BR , *et al* Dual nature of the adaptive immune system in lampreys. Nature 2009; 459: 796–801. 19474790 10.1038/nature08068 PMC2714547

[13] Alder MN , Herrin BR , Sadlonova A , *et al* Antibody responses of variable lymphocyte receptors in the lamprey. Nat Immunol 2008; 9: 319–327. 18246071 10.1038/ni1562

[14] Pancer Z , Amemiya CT , Ehrhardt GR , Ceitlin J , Gartland GL , Cooper MD . Somatic diversification of variable lymphocyte receptors in the agnathan sea lamprey. Nature 2004; 430: 174–180. 15241406 10.1038/nature02740

[15] Holland SJ , Gao M , Hirano M , *et al* Selection of the lamprey VLRC antigen receptor repertoire. Proc Natl Acad Sci USA 2014; 111: 14834–14839. 25228760 10.1073/pnas.1415655111 PMC4205635

[16] Das S , Li J , Holland SJ , *et al* Genomic donor cassette sharing during VLRA and VLRC assembly in jawless vertebrates. Proc Natl Acad Sci USA 2014; 111: 14828–14833. 25228758 10.1073/pnas.1415580111 PMC4205630

[17] Nutt SL , Hodgkin PD , Tarlinton DM , Corcoran LM . The generation of antibody‐secreting plasma cells. Nat Rev Immunol 2015; 15: 160–171. 25698678 10.1038/nri3795

[18] Hawkins ED , Turner ML , Wellard CJ , Zhou JH , Dowling MR , Hodgkin PD . Quantal and graded stimulation of B lymphocytes as alternative strategies for regulating adaptive immune responses. Nat Commun 2013; 4: 2406. 24009041 10.1038/ncomms3406 PMC3778729

[19] Mitchell S , Roy K , Zangle TA , Hoffmann A . Nongenetic origins of cell‐to‐cell variability in B lymphocyte proliferation. Proc Natl Acad Sci USA 2018; 115: E2888–E2897. 29514960 10.1073/pnas.1715639115 PMC5866559

[20] Hawkins ED , Markham JF , McGuinness LP , Hodgkin PD . A single‐cell pedigree analysis of alternative stochastic lymphocyte fates. Proc Natl Acad Sci USA 2009; 106: 13457–13462. 19633185 10.1073/pnas.0905629106 PMC2715326

[21] Hawkins ED , Turner ML , Dowling MR , van Gend C , Hodgkin PD . A model of immune regulation as a consequence of randomized lymphocyte division and death times. Proc Natl Acad Sci USA 2007; 104: 5032–5037. 17360353 10.1073/pnas.0700026104 PMC1821128

[22] Heinzel S , Binh Giang T , Kan A , *et al* A Myc‐dependent division timer complements a cell‐death timer to regulate T cell and B cell responses. Nat Immunol 2017; 18: 96–103. 27820810 10.1038/ni.3598

[23] Turner ML , Hawkins ED , Hodgkin PD . Quantitative regulation of B cell division destiny by signal strength. J Immunol 2008; 181: 374–382. 18566403 10.4049/jimmunol.181.1.374

[24] Tassia MG , Whelan NV , Halanych KM . Toll‐like receptor pathway evolution in deuterostomes. P Natl Acad Sci USA 2017; 114: 7055–7060. 10.1073/pnas.1617722114 PMC5502590 28630328

[25] Satake H , Sekiguchi T . Toll‐like receptors of deuterostome invertebrates. Front Immunol 2012; 3. 22566918 10.3389/fimmu.2012.00034 PMC3342246

[26] Nie L , Cai SY , Shao JZ , Chen J . Toll‐like receptors, associated biological roles, and signaling networks in non‐mammals. Front Immunol 2018; 9. 30034391 10.3389/fimmu.2018.01523 PMC6043800

[27] Hartl M , Glasauer S , Valovka T , Breuker K , Hobmayer B , Bister K . Hydra myc2, a unique pre‐bilaterian member of the myc gene family, is activated in cell proliferation and gametogenesis. Biol Open 2014; 3: 397–407. 24771621 10.1242/bio.20147005 PMC4021362

[28] Hartl M , Mitterstiller AM , Valovka T , Breuker K , Hobmayer B , Bister K . Stem cell‐specific activation of an ancestral myc protooncogene with conserved basic functions in the early metazoan *Hydra* . Proc Natl Acad Sci USA 2010; 107: 4051–4056. 20142507 10.1073/pnas.0911060107 PMC2840113

[29] Hengartner MO , Horvitz HR . C. elegans cell survival gene ced‐9 encodes a functional homolog of the mammalian proto‐oncogene bcl‐2. Cell 1994; 76: 665–676. 7907274 10.1016/0092-8674(94)90506-1

[30] Haecker G , Vaux DL . Viral, worm and radical implications for apoptosis. Trends Biochem Sci 1994; 19: 99–100. 8203023 10.1016/0968-0004(94)90197-x

[31] Duffy KR , Wellard CJ , Markham JF , *et al* Activation‐induced B cell fates are selected by intracellular stochastic competition. Science 2012; 335: 338–341. 22223740 10.1126/science.1213230

[32] Hodgkin PD . Modifying clonal selection theory with a probabilistic cell. Immunol Rev 2018; 285: 249–262. 30129201 10.1111/imr.12695 PMC6446824

[33] Martin FA , Morata G . Compartments and the control of growth in the *Drosophila* wing imaginal disc. Development 2006; 133: 4421–4426. 17035294 10.1242/dev.02618

[34] Beira JV , Paro R . The legacy of *Drosophila* imaginal discs. Chromosoma 2016; 125: 573–592. 27153833 10.1007/s00412-016-0595-4 PMC5023726

[35] Gallant P . Myc, cell competition, and compensatory proliferation. Cancer Res 2005; 65: 6485–6487. 16061622 10.1158/0008-5472.CAN-05-1101

[36] Grifoni D , Bellosta P . *Drosophila* Myc: a master regulator of cellular performance. Biochim Biophys Acta 2015; 1849: 570–581. 25010747 10.1016/j.bbagrm.2014.06.021 PMC4287454

[37] Sato T , Vries RG , Snippert HJ , *et al* Single Lgr5 stem cells build crypt‐villus structures *in vitro* without a mesenchymal niche. Nature 2009; 459: 262–265. 19329995 10.1038/nature07935

[38] Kim MJ , Xia B , Suh HN , *et al* PAF‐Myc‐controlled cell stemness is required for intestinal regeneration and tumorigenesis. Dev Cell 2018; 44: e584. 10.1016/j.devcel.2018.02.010 PMC5854208 29533773

[39] Finch AJ , Soucek L , Junttila MR , Swigart LB , Evan GI . Acute overexpression of Myc in intestinal epithelium recapitulates some but not all the changes elicited by Wnt/beta‐catenin pathway activation. Mol Cell Biol 2009; 29: 5306–5315. 19635809 10.1128/MCB.01745-08 PMC2747972

[40] Liu C , Banister CE , Weige CC , *et al* PRDM1 silences stem cell‐related genes and inhibits proliferation of human colon tumor organoids. Proc Natl Acad Sci USA 2018; 115: E5066–E5075. 29760071 10.1073/pnas.1802902115 PMC5984534

[41] van der Heijden M , Zimberlin CD , Nicholson AM , *et al* Bcl‐2 is a critical mediator of intestinal transformation. Nat Commun 2016; 7: 10916. 26956214 10.1038/ncomms10916 PMC4786877

[42] Strand MR . The insect cellular immune response. Insect Sci 2008; 15: 1–14.

[43] Carton Y , Poirie M , Nappi AJ . Insect immune resistance to parasitoids. Insect Sci 2008; 15: 67–87.

[44] Williams MJ . Drosophila hemopoiesis and cellular immunity. J Immunol 2007; 178: 4711–4716. 17404248 10.4049/jimmunol.178.8.4711

[45] Anderl I , Vesala L , Ihalainen TO , *et al* Transdifferentiation and proliferation in two distinct hemocyte lineages in *Drosophila melanogaster* larvae after wasp infection. PLoS Pathog 2016; 12: e1005746. 27414410 10.1371/journal.ppat.1005746 PMC4945071

[46] Zettervall CJ , Anderl I , Williams MJ , *et al* A directed screen for genes involved in Drosophila blood cell activation. Proc Natl Acad Sci USA 2004; 101: 14192–14197. 15381778 10.1073/pnas.0403789101 PMC521135

[47] Asha H , Nagy I , Kovacs G , Stetson D , Ando I , Dearolf CR . Analysis of Ras‐induced overproliferation in *Drosophila* hemocytes. Genetics 2003; 163: 203–215. 12586708 10.1093/genetics/163.1.203 PMC1462399

[48] Sinenko SA , Mathey‐Prevot B . Increased expression of *Drosophila* tetraspanin, Tsp68C, suppresses the abnormal proliferation of ytr‐deficient and Ras/Raf‐activated hemocytes. Oncogene 2004; 23: 9120–9128. 15480416 10.1038/sj.onc.1208156

[49] Schmucker D , Chen B . Dscam and DSCAM: complex genes in simple animals, complex animals yet simple genes. Genes Dev 2009; 23: 147–156. 19171779 10.1101/gad.1752909

[50] Watson FL , Puttmann‐Holgado R , Thomas F , *et al* Extensive diversity of Ig‐superfamily proteins in the immune system of insects. Science 2005; 309: 1874–1878. 16109846 10.1126/science.1116887

[51] Dong Y , Taylor HE , Dimopoulos G . AgDscam, a hypervariable immunoglobulin domain‐containing receptor of the *Anopheles gambiae* innate immune system. PLoS Biol 2006; 4: e229. 16774454 10.1371/journal.pbio.0040229 PMC1479700

[52] Boyden SV . Natural antibodies and the immune response. Adv Immunol 1966; 5: 1–28. 5332818 10.1016/s0065-2776(08)60271-0

[53] Hodgkin PD . An antigen valence theory to explain the evolution and organization of the humoral immune response. Immunol Cell Biol 1997; 75: 604–618. 9492200 10.1038/icb.1997.95

[54] Bernasconi NL , Onai N , Lanzavecchia A . A role for Toll‐like receptors in acquired immunity: up‐regulation of TLR9 by BCR triggering in naive B cells and constitutive expression in memory B cells. Blood 2003; 101: 4500–4504. 12560217 10.1182/blood-2002-11-3569

[55] Mesin L , Ersching J , Victora GD . Germinal center B cell dynamics. Immunity 2016; 45: 471–482. 27653600 10.1016/j.immuni.2016.09.001 PMC5123673

[56] Zhang Y , Garcia‐Ibanez L , Toellner KM . Regulation of germinal center B‐cell differentiation. Immunol Rev 2016; 270: 8–19. 26864101 10.1111/imr.12396 PMC4755139

[57] Finkin S , Hartweger H , Oliveira TY , Kara EE , Nussenzweig MC . Protein amounts of the MYC transcription factor determine germinal center B cell division capacity. Immunity 2019; 51: e325. 10.1016/j.immuni.2019.06.013 PMC6703930 31350178

[58] Dominguez‐Sola D , Victora GD , Ying CY , *et al* The proto‐oncogene MYC is required for selection in the germinal center and cyclic reentry. Nat Immunol 2012; 13: 1083–1091. 23001145 10.1038/ni.2428 PMC3711534

[59] Bryant VL , Hodgkin PD . Life, death, and antibodies. Science 2017; 358: 171–172. 29026032 10.1126/science.aap8728

[60] Tas JM , Mesin L , Pasqual G , *et al* Visualizing antibody affinity maturation in germinal centers. Science 2016; 351: 1048–1054. 26912368 10.1126/science.aad3439 PMC4938154

[61] Mayer CT , Gazumyan A , Kara EE , *et al* The microanatomic segregation of selection by apoptosis in the germinal center. Science 2017; 358. 10.1126/science.aao2602 PMC5957278 28935768

[62] Vikstrom I , Tarlinton DM . B cell memory and the role of apoptosis in its formation. Mol Immunol 2011; 48: 1301–1306. 21144588 10.1016/j.molimm.2010.10.026

[63] Bannard O , Horton RM , Allen CD , An J , Nagasawa T , Cyster JG . Germinal center centroblasts transition to a centrocyte phenotype according to a timed program and depend on the dark zone for effective selection. Immunity 2013; 39: 912–924. 24184055 10.1016/j.immuni.2013.08.038 PMC3828484

